# A new deep-sea species of *Flabelligena* from off the South Orkney Islands, the Southern Ocean

**DOI:** 10.3897/BDJ.8.e53312

**Published:** 2020-06-08

**Authors:** Naoto Jimi, Akito Ogawa, Shimpei F Hiruta, Minoru Ikehara, Satoshi Imura

**Affiliations:** 1 National Institute of Polar Research, 10-3 Midoricho, Tachikawa, Tokyo, Japan National Institute of Polar Research, 10-3 Midoricho Tachikawa, Tokyo Japan; 2 Graduate School of Science, The University of Tokyo, 7-3-1 Hongo, Bunkyo-ku, Tokyo, Japan Graduate School of Science, The University of Tokyo, 7-3-1 Hongo Bunkyo-ku, Tokyo Japan; 3 Center for Molecular Biodiversity Research, National Museum of Nature and Science, 4-1-1 Amakubo, Tsukuba, Ibaraki, Japan Center for Molecular Biodiversity Research, National Museum of Nature and Science, 4-1-1 Amakubo Tsukuba, Ibaraki Japan; 4 Center for Advanced Marine Core Research, Kochi University, Monobe-otsu 200 Nankoku, Kochi, Japan Center for Advanced Marine Core Research, Kochi University, Monobe-otsu 200 Nankoku Kochi Japan; 5 National Institute of Polar Research, 10-3 Midoricho, Tachikawa, Japan National Institute of Polar Research, 10-3 Midoricho Tachikawa Japan; 6 Department of Polar Science, SOKENDAI (The Graduate University for Advanced Studies), 10-3 Midoricho, Tachikawa, Tokyo, Japan Department of Polar Science, SOKENDAI (The Graduate University for Advanced Studies), 10-3 Midoricho Tachikawa, Tokyo Japan

**Keywords:** Antarctica, Southern Ocean, Polychaeta, *Hakuho maru*

## Abstract

**Background:**

A new acrocirrid species, *Flabelligena hakuhoae* **sp. nov.**, is described from off the South Orkney Islands, the Southern Ocean. Individuals of the new species were collected by rock dredging, 2036–2479 m in depth.

**New information:**

The new species can be distinguished from its congeners by the number of branchiae, position and length of paired ventral large papillae and length of body papillae.

## Introduction

Acrocirridae Banse, 1969 (clade Cirratuliformia) consists of 43 species in nine genera distributed from the intertidal zone to the deep seafloor (Magalhães and Bailey-Brock 2011, Martínez et al. 2019). They are found in sandy areas and under rocks, but some genera are pelagic (Osborn et al. 2009).

*Flabelligena* Gillet, 2001 belongs to the Acrocirridae. It has a minute body, body papillae, spinulose or serrated (not cross-barred) notochaetae, 1–3 pairs of branchiae and a pair of frontal palps (Aguirrezabalaga and Ceberio 2006). They live in sandy areas, mainly in bathyal to abyssal depths. The six described *Flabelligena* species are known mainly from the North Atlantic Ocean, three of which are known from the Southwest Atlantic, Mediterranean and South Indian Oceans (Aguirrezabalaga and Ceberio 2006). Several polychaetes faunal surveys have been carried out around the Southern Ocean (e.g. Hartman 1953, Hartman 1964, Hartman 1966, Hartman 1967, Monro 1939). However, with the exception of *Flabelligena amoureuxi* Gillet, 2001 from off the Crozet Island (South Indian Ocean) and *F. erratica* (Orensanz, 1974) from off Falkland Islands (South Atlantic Ocean) (Gillet 2001, Orensanz 1974), there are no records of *Flabelligena* from around the Southern Ocean.

During the research cruise KH19-06-Leg4 by R/V *Hakuho maru*, the first author found individuals of *Flabelligena* from off the South Orkney Islands. In this paper, we describe the specimens as a new species.

## Materials and methods

**Figure 1.**
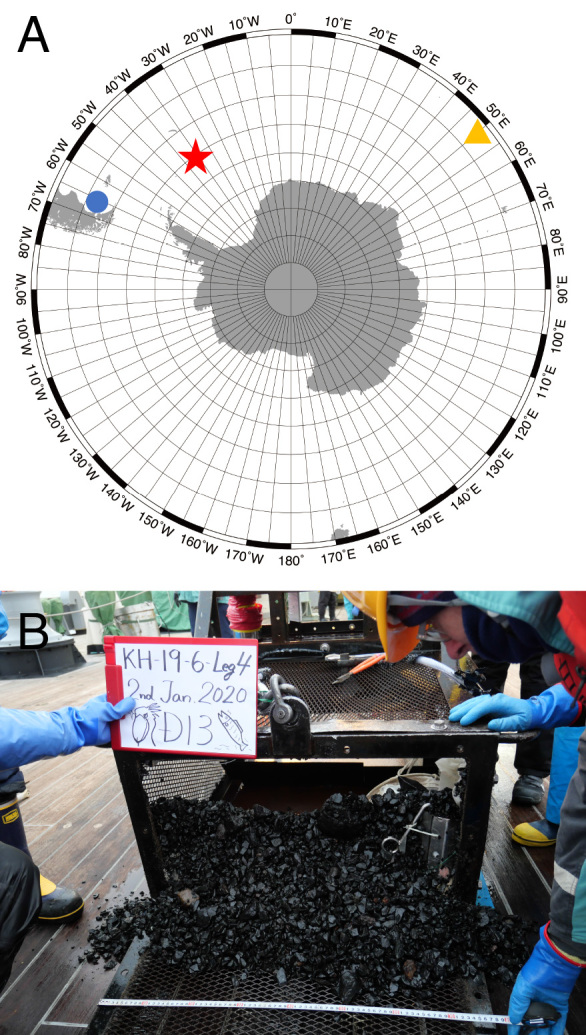
Sampling sites and sediments of the type locality. **A.** Type localities of three *Flabelligena* species from the southern hemisphere: red star, *F. hakuhoae* sp. nov. in this study; blue circle, *F. erratica* in Orensanz (1974); yellow triangle, *F. amoureuxi* in Gillet (2001); **B.** The collection gear after sampling at the type locality (red star in A), off the South Orkney Islands, the Southern Ocean.

Specimens were collected from off the South Orkney Islands (Fig. 1A), the Southern Ocean (60°33.54'S, 35°24.43'W–60°34.07'S, 35°23.40'W), 2036 – 2479 m in depth by a rock dredge (Fig. 1B) and extracted from the rock and silt sediments using a 32 μm sieve with seawater and fixed in 70% ethanol. After preservation, these specimens were observed with Nikon SMZ18 and Nikon ECLIPSE 80i microscopes and photographed with a Nikon D5200 digital camera. The paratype specimen was washed in a phosphate-buffered saline solution and dehydrated in a graded ethanol series, dried in a critical-point dryer (HITACHI HCP-1) using liquid CO_2_ and coated with gold in an ion sputter (HITACHI E-1045) for SEM observations. Observations were conducted using an SEM instrument (HITACHI S-3000N). Type material is deposited in the National Museum of Nature and Science, Tsukuba (**NSMT**) and Invertebrate Collection of the Hokkaido University Museum (**ICHUM**).

We followed the morphological terminology of Aguirrezabalaga and Ceberio (2006) and Martínez et al. (2019) in the taxonomic description below.

## Taxon treatments

### Flabelligena hakuhoae

Jimi sp. n.

9297C098-C02D-5779-918F-9DA3AD471734

urn:lsid:zoobank.org:act:9E6C4840-0D03-4A24-B825-AC4EADD0CC8B

#### Materials

- **Type status:** Holotype. **Taxon:** phylum: Annelida; family: Acrocirridae; genus: Flabelligena; **Location:** higherGeography: Southern Ocean; off the South Orkney Islands; waterBody: Southern Ocean; locality: off the South Orkney Islands; verbatimDepth: 2036-2479 m; **Identification:** identifiedBy: Naoto Jimi; **Event:** samplingProtocol: rock dredge; eventDate: 02-01-2020; year: 2020; month: 1; day: 2; habitat: rocks and sands; **Record Level:** language: en; ownerInstitutionCode: NSMT
- **Type status:** Paratype. **Taxon:** phylum: Annelida; family: Acrocirridae; genus: Flabelligena; **Location:** higherGeography: Southern Ocean; off the South Orkney Islands; waterBody: Southern Ocean; locality: off the South Orkney Islands; verbatimDepth: 2036-2479 m; **Identification:** identifiedBy: Naoto Jimi; **Event:** samplingProtocol: rock dredge; eventDate: 02-01-2020; year: 2020; month: 1; day: 2; habitat: rocks and sands; **Record Level:** language: en; ownerInstitutionCode: ICHUM

#### Description

**Figure 2.**
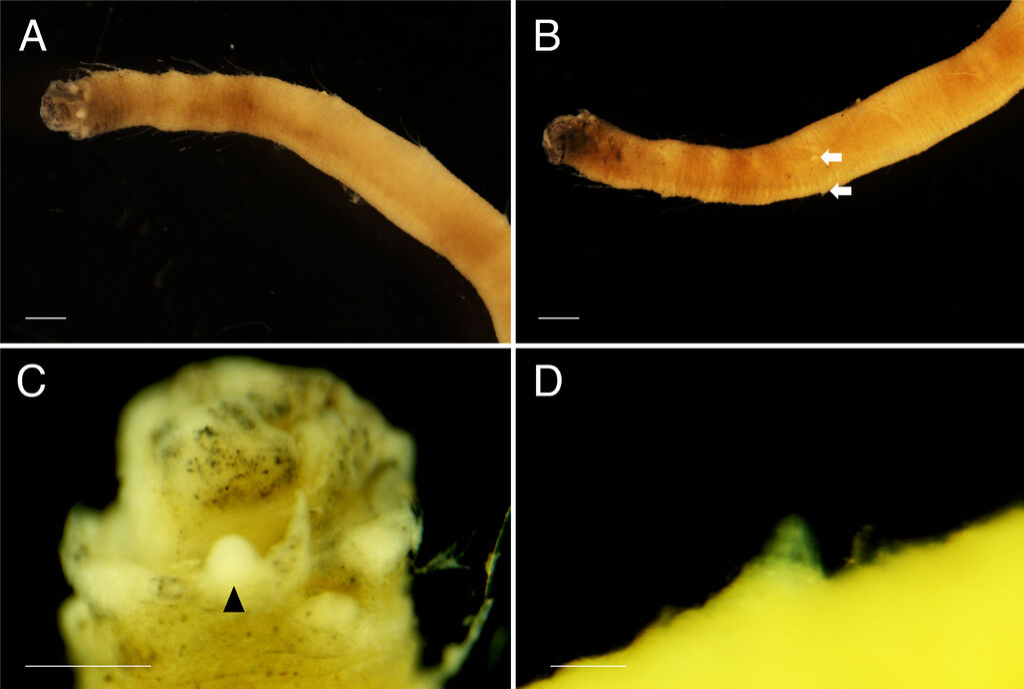
*Flabelligena hakuhoae* sp. nov., holotype (NSMT-Pol H-813). **A.** Anterior end, dorsal view; **B.** anterior end, ventral view, arrows indicate ventral papillae; **C.** anterior end, dorsal enlarged view, an arrow head indicates a branchia; **D.** ventral large papilla, enlarged view. Scale bars: A–B, 500 μm; C, 125 μm; D, 50 μm.

**Figure 3.**
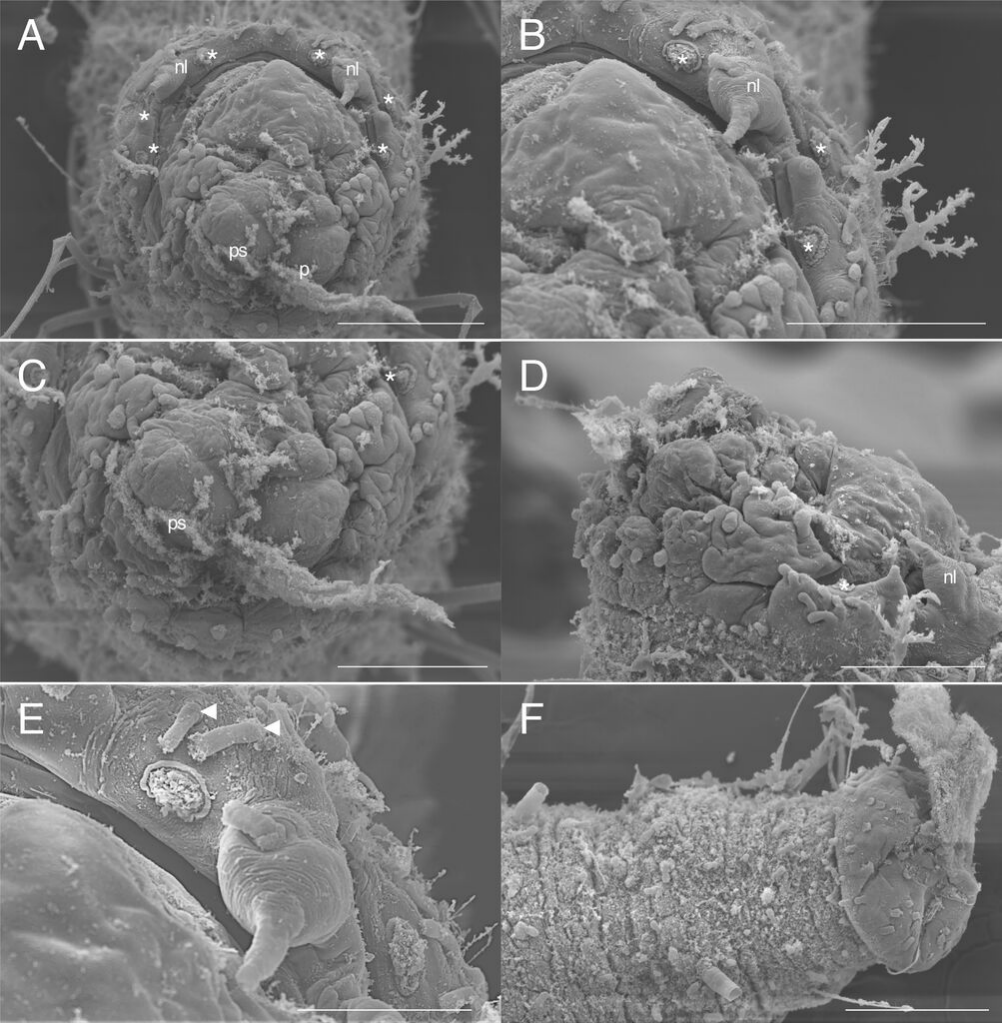
*Flabelligena hakuhoae* sp. nov., paratype (ICHUM-6113), scanning electron micrographs. **A.** Anterior end, frontal view, proboscis is not everted; **B.** Anterior end, dorsal side, frontal view; **C.** Anterior end, ventral side, frontal view; **D.** Anterior end, lateral view; **E.** branchial scars and nephridial lobe, enlarged view; **F.** pygidium, dorsal view. Asterisks indicate branchial scars. Arrow heads indicate papillae around branchial scars. Abbreviations: nl, nephridial lobe; p, palp; ps, palp scar. Scale bars: A, 300 μm; B–D, 200 μm; E 100 μm; F, 200 μm.

**Holotype (NSMT-Pol H-813)** 1.8 cm long, 1 mm wide (without chaetae, at widest chaetiger) for 27 chaetigers (incomplete). Body cylindrical (Fig. 2), rounded in anterior and posterior end, yellowish in life and after fixation, with darker pigmentation around the anterior end, without ventral centre line, surface papillated, slightly inflated laterally in chaetigers 5–11, subannulations absent. Body papillae presented on several areas. Papillae of body surface oval, each papilla about 15 μm long and 10 μm wide (n = 10), not arranged in transverse rows, scattered. Papillae around branchial scars (Fig. 3) cylindrical, each papilla about 35 μm long and 10 μm wide (n = 10), 5–6 papillae present around branchial scars. Papillae on prostomium (Fig. 3A), cylindrical, each papilla about 35 μm long and 20 μm wide (n = 10), scattered. Papillae between noto- and neurochaetae (Fig. 5A and D), circular, each papilla about 30 μm long and 30 μm wide (n = 10), one papilla between chaetae (Fig. 5D). Paired ventral large papillae present ventral side of parapodium in chaetiger 6 (Fig. 2B and D), short (72 μm long and 55 μm wide), conical, one pair.

**Figure 4.**
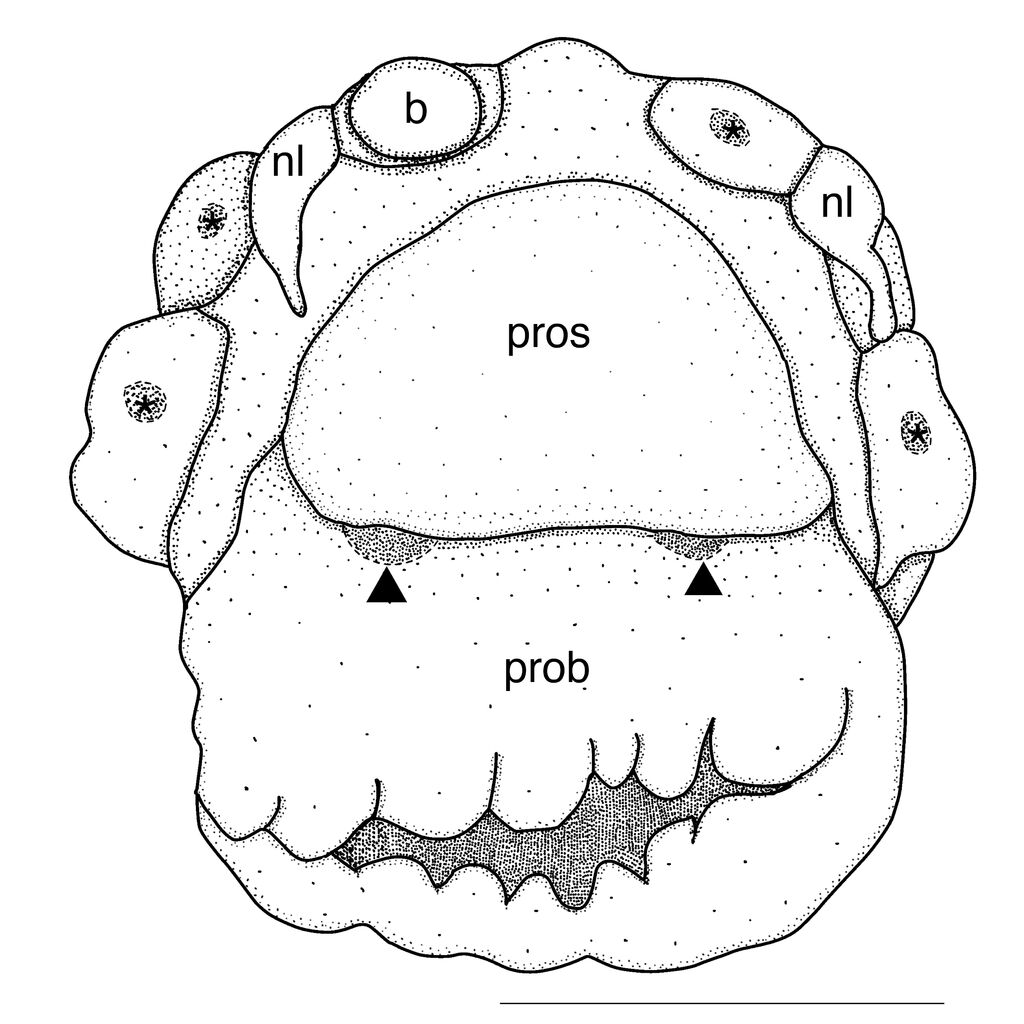
*Flabelligena hakuhoae* sp. nov., holotype (NSMT-Pol H-813), anterior end, frontal view. Proboscis is everted. Abbreviation: b, branchia; nl, nephridial lobe; prob, proboscis; pros, prostomium. Asterisks indicate branchial scars. Arrow heads indicate palp scars. Scale bar = 500 μm.

**Figure 5.**
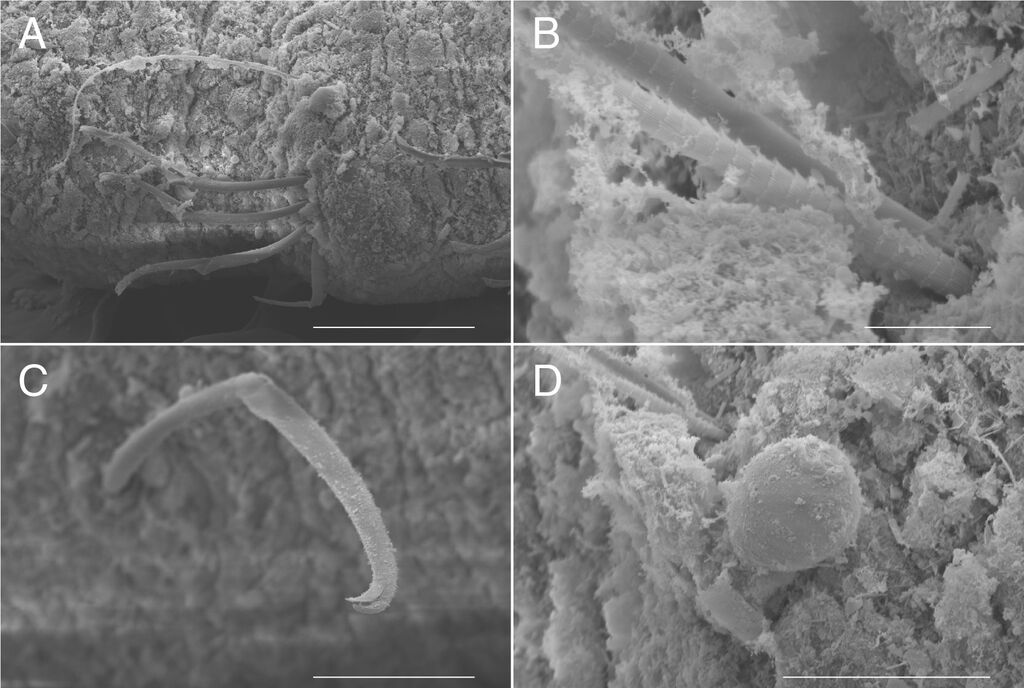
*Flabelligena hakuhoae* sp. nov., paratype (ICHUM-6113), scanning electron micrographs. **A.** Notochaetae and neurochaetae, posterior region; **B.** Notochaetae; **C.** Neurochaetae; **D.** Body papilla under the notochaetae. Scale bars: A, 200 μm; B, 10 μm; C, 100 μm; D, 50 μm.

Prostomium subtriangular, eyes absent, nuchal organs absent. Peristomium distinct. Palp scars on anterior margin of prostomium, one pair (Figs 3, 4), palps cirrigerous shape, with numerous cirri on ventral side. Branchial scars three pairs (Figs 3, 4), branchiae globular shape (Figs 2, 4). Nephridial lobe consists of nephriodiopore papillae and globular base (Fig. 3B), two nephriodiopore papillae per globular base (Fig. 3B). Notochaetae spinulose capillary (Fig. 5B), 1–2 per fascicle throughout the body. Neurochaetae compound (Fig. 5C): blades sickle-shaped, bidentate (one over another), inner edge of blade smooth; blade length 6/5 of shaft in chaetigers 1–17, as long as shaft in chaetigers 18–27; 1–2 per fascicle in chaetigers 1–17, 3–4 per fascicle in chaetigers 18–27. Pygidium lost.

**Paratype (ICHUM-6113, used in SEM observation)** 1.2 cm in length, 0.9 mm in width (without chaetae, at widest chaetiger), 23 chaetigers. Pygidium rounded (Fig. 3F), with many body papillae (about 15 μm long and 8 μm wide, n = 10), without pygidial cirrus. Other characters the same as holotype.

#### Etymology

The species is named after the R/V *Hakuho-maru*, a gear of the ship which collected the type specimens from the Southern Ocean. The specific name is a noun in the genitive case.

#### Distribution

The new species is only known from the type locality, off the South Orkney Islands, the Southern Ocean (60°33.54'S, 35°24.43'W–60°34.07'S, 35°23.40'W), 2036 – 2479 m in depth.

#### Taxon discussion

*Flabelligena hakuhoae* **sp. nov.** belongs to *Flabelligena* because it has the following features: retractile anterior region, absence of distinct body region, presence of various pairs of branchiae, composite falcigerous neurochaetae, simple spinulose or serrated notochaetae (Aguirrezabalaga and Ceberio 2006). This species is different from the other known species by having the following features (see Table 1): i) paired ventral large papillae present on the ventral side of parapodium in chaetiger 6; ii) three pairs of branchiae; iii) short body papillae between noto- and neurochaetae. *Flabelligena erratica* (Orensanz, 1974) and *F. gascognensis* Aguirrezabalaga and Ceberio, 2006 have paired ventral papillae between chaetigers 6–7, but do not have them at the ventral side of a parapodium in chaetiger 6 (Orensanz 1974, Aguirrezabalaga and Ceberio 2006). Additionally, *F. gascognensis* has long (as long as neurochaetae) papillae around the parapodium, while the new species does not have them (1/6 of neurochaetae). Two species, *F. amoureuxi* Gillet, 2001 and *F. erratica*, have been found in the vicinity of the Southern Ocean as well as this new one. The new species can be distinguished from the other two species by having three pairs of branchiae, paired ventral large papillae near the parapodium of chaetiger 6 and absence of eyes. *Flabelligena amoureuxi* has a pair of branchiae and does not have paired ventral large papillae and eyes. *Flabelligena erratica* has a pair of branchiae, paired ventral large papillae between chaetigers 6–7 and eyes (see Table 1).

## Supplementary Material

XML Treatment for Flabelligena hakuhoae

## Figures and Tables

**Table 1.** A comparative table of *Flabelligena* (modified from Aguirrezabalaga and Ceberio 2006). The morphological features are based on original descriptions and figures.

	***F. cirrata*** **Hartman and Fauchald 1971**	***F. amoureuxiGillet 2001***	***F. erratica*** **Orensanz 1974**	***F. biscayensis*** **Kolmer 1985**	***F. mediterranea*** **Kolmer 1985**	***F. gascognensis*** **Aguirrezabalaga and Ceberio 2006**	***F. hakuhoae*** **sp. nov.** **This study**
**Pair of branchiae**	1	1	1	2	2	3	3
**Pair of ventral large papillae**	absent	absent	one pair, between chaetigers 6–7	absent	one pair, between chaetigers 4–5	one pair, between chaetigers 6–7	one pair, ventral side of parapodium in chaetiger 6
**Chaetigers**	17–24	30	20–30	incomplete (11)	incomplete (8)	incomplete (25)	23–?
**Notochaetae**	serrated 1–2	spinulose 1	spinulose 1	spinulose 2–5	spinulose 2–5	spinulose ?–4	spinulose 1–2
**Neurochaetae**	composite 4–8	composite 3–5	composite 1–3	composite 1	composite 2–4	composite 2–4	composite 1–4
**Eyes**	0	0	2	0	0	0	0
**Papillae between noto- and neurochaetae**	short, 2/9 of neurochaetae	short, 4/15 of neurochaetae	short, 3/8 of neurochaeta	long, as long as neurochaetae	long, as long as neurochaetae	long, as long as neurochaetae	short, 1/6 of neurochaetae
**Type locality**	New England (NW Atlantic)	Crozet Islands (Indian Ocean)	Argentina (SW Atlantic)	Bay of Biscay (NE Atlantic)	Mediterranean Sea	Bay of Biscay (NE Atlantic)	off the South Orkney Islands (Southern Ocean)
**Depth (m)**	466–530	215–980	288	2210	4690	545–1113	2036–2479
